# Effect of Black Corn Anthocyanin-Rich Extract (*Zea mays* L.) on Cecal Microbial Populations In Vivo (*Gallus gallus*)

**DOI:** 10.3390/nu14214679

**Published:** 2022-11-04

**Authors:** Thaisa Agrizzi Verediano, Nikita Agarwal, Hércia Stampini Duarte Martino, Nikolai Kolba, Mariana Grancieri, Maria Cristina Dias Paes, Elad Tako

**Affiliations:** 1 Nutrition and Health Department, Universidade Federal de Viçosa, Vicosa 36571-000, Minas Gerais, Brazil; 2 Department of Food Science, Cornell University, Stocking Hall, Ithaca, NY 14853, USA; 3 Empresa Brasileira de Pesquisa e Agropecuária (EMBRAPA), Sete Lagoas 35701-970, Minas Gerais, Brazil

**Keywords:** cyanidin, intestinal barrier, phenolic components, goblet cells

## Abstract

Black corn has been attracting attention to investigate its biological properties due to its anthocyanin composition, mainly cyanidin-3-glucoside. Our study evaluated the effects of black corn extract (BCE) on intestinal morphology, gene expression, and the cecal microbiome. The BCE intra-amniotic administration was evaluated by an animal model in *Gallus gallus*. The eggs (*n* = 8 per group) were divided into: (1) no injection; (2) 18 MΩ H_2_O; (3) 5% black corn extract (BCE); and (4) 0.38% cyanidin-3-glucoside (C3G). A total of 1 mL of each component was injected intra-amniotic on day 17 of incubation. On day 21, the animals were euthanized after hatching, and the duodenum and cecum content were collected. The cecal microbiome changes were attributed to BCE administration, increasing the population of *Bifidobacterium* and *Clostridium,* and decreasing *E. coli.* The BCE did not change the gene expression of intestinal inflammation and functionality. The BCE administration maintained the villi height, Paneth cell number, and goblet cell diameter (in the villi and crypt), similar to the H_2_O injection but smaller than the C3G. Moreover, a positive correlation was observed between *Bifidobacterium*, *Clostridium*, *E. coli*, and villi GC diameter. The BCE promoted positive changes in the cecum microbiome and maintained intestinal morphology and functionality.

## 1. Introduction

Corn, also known as maize (*Zea mays* L.), is one of the most produced cereals and one of the major food sources worldwide [1]. In recent decades, scientific research has focused on pigmented corn varieties due to their beneficial health properties [2]. Among them, black corn (*Zea mays* spp.) is a variety traditionally cultivated in South and Central America that has an affinity for warm and dry climates [1]. Black and purple corn can accumulate anthocyanin in different tissues; thus, these varieties have a significant concentration of these flavonoids [3].

Anthocyanins are bioactive water-soluble pigments observed in nature, mainly in the form of glycosides, providing color in plants, fruits, vegetables, and flowers [4]. Extract from anthocyanin-rich foods has been reported to have health properties [5], as anti-inflammatory [6], antioxidant [7], gut microbiota modulation [8], improving cholesterol [9], and glucose metabolism [10]. Seven hundred anthocyanin structures have been identified in nature; however, some of them are verified in higher concentrations in plants, as cyanidin, delphinidin, malvidin, pelargonidin, peonidin, and petunidin [4,11]. Among them, cyanidin-3-glucoside (C3G) is the most predominant anthocyanin naturally observed in plants [12]. C3G is the main anthocyanin observed in the black corn flour composition (30.40 mg/100 g); however, a high concentration of phenolic components (614.30 mg GAE—gallic acid equivalent/100 g) is also observed in the food matrix [13].

C3G has an *O*-glycosylated anthocyanin with two hydroxyls on the third aromatic ring, which confers vigorous antioxidant activity [14]. The leading site of C3G catabolism is the small intestine, in which the C3G molecules are hydrolyzed to aglycones and degraded to specific phenolic compounds such as protocatechuic acid, phloroglucinaldehyde, vanillic acid, and ferulic acid by the gut microbiota [12]. Intestinal microbiota are able to utilize phenolic compounds as a substrate to obtain energy and to create fermentable metabolites with biological functions [15]. The metabolites produced from the intestinal metabolism of C3G inhibit inflammatory pathways such as nuclear factor-kappa B (Nf-κB) [14]. Since the intestinal tract acts as a barrier against external pathogens [16], C3G and its metabolites contribute to maintaining the intestinal barrier integrity, mucosal barrier, and microbiota composition [12,17].

Recently, we demonstrated that black corn soluble extract, composed of soluble fiber and phenolic compounds, promoted goblet cell proliferation and upregulated biomarkers related to the epithelial intestinal integrity pathway such as AMP—activated protein kinase (AMPK) and caudal-related homeobox transcriptional factor 2 (CDX2) [18]. Previously, it was suggested that fermented soluble extract promotes the proliferation of beneficial gut bacteria, which affects intestinal brush border membrane morphology, including the growth of villus and crypt and goblet cell proliferation [19]. However, the effects of the isolated phenolic extract on the morphology and gut microbiota might differ [20]. Saffron flower, a source of polyphenols, showed an unfavorable effect on the microbial population, brush border morphology, and functionality [21]. Depending on their dosage, polyphenols can have negative impacts due to interference with nutrient metabolism [20]. Resveratrol and pterostilbene (5%) did not promote modification in the taxonomy of the cecal microbiota but increased morphological changes [22]. For this reason, phenolics and other bioactive compounds in food sources and byproducts need to be quantified and their biological effects and safety validated in living organisms.

The intra-amniotic (in ovo) approach is widely accepted for assessing potential effects of bioactive components [18,21,23,24,25]. The in ovo technique in *Gallus gallus* allows the administration of components into the amniotic fluid. Therefore, as the embryo consumes the amniotic fluid before hatching, the biological changes after hatching are a predictor of the effects of the bioactive component administered [19]. Considering the possible consumption of bioactive compound-rich extracts, it is relevant to assess the biological effects of dried extracts to validate their application. Since anthocyanin-rich extract from black corn has not been explored for intestinal health so far, this experiment was carried out to investigate the impact of intra-amniotic administration of black corn anthocyanin-rich extract on the intestinal brush border membrane functionality, morphology, and cecal microbial populations.

## 2. Materials and Methods

### 2.1. Materials

Black corn grains (TO002) were provided by the Brazilian Agriculture Research Corporation (EMBRAPA) from the Maize Germplasm Bank of the Maize and Sorghum National Research Center (Sete Lagoas, MG, Brazil). Cyanidin-3-glucoside chloride (>98%) was obtained from Sigma-Aldrich^®^ (Cat # PHL89616, St. Louis, MO, USA).

### 2.2. Black Corn Extract Procedure

**Figure 1:**
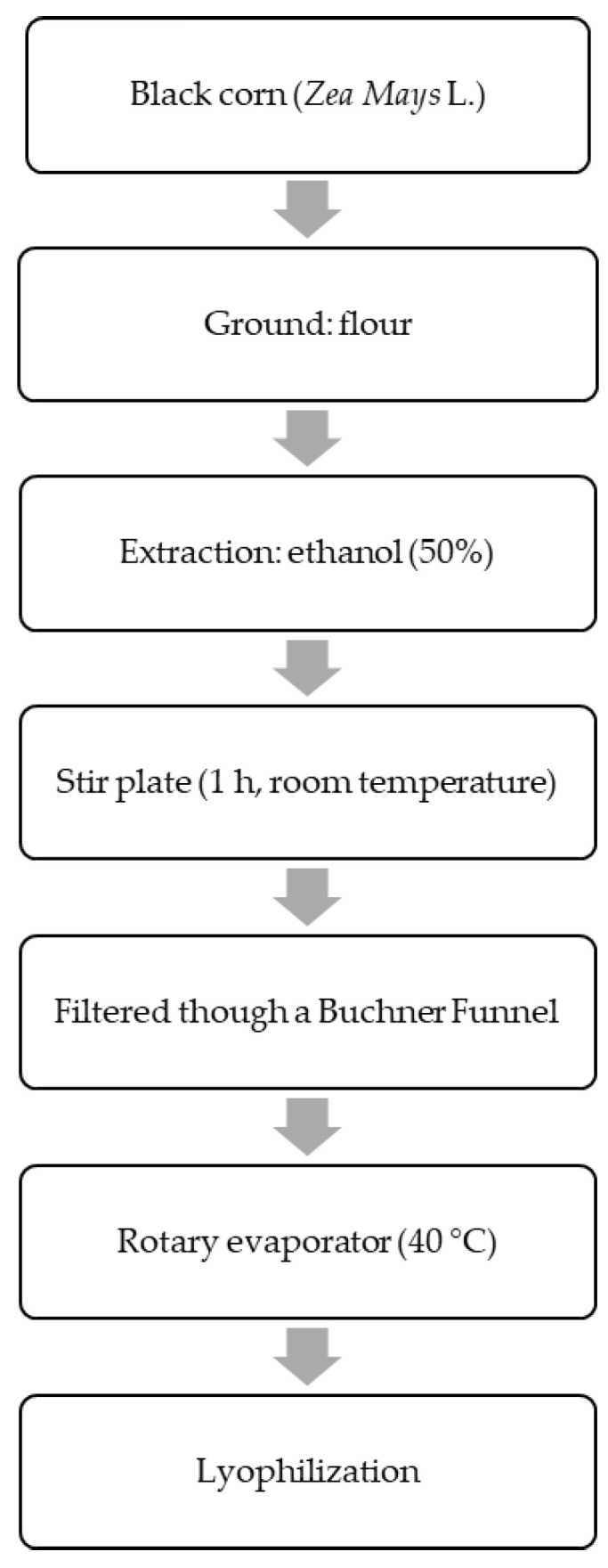
Flowchart of the black corn extract procedure.

Prior to extraction, black corn grains were ground with a 1.0 mm stainless steel sieve (Willy, Solab^®,^ Piracicaba, Brazil) to prepare the corn flour. The production of the extract was performed at room temperature without any light. The black corn flour was added to ethanol 50% (1:10 *v*/*v*), then submitted to a magnetic stir plate (100 rpm/60 min/room temperature). After the allotted time had passed, the suspension was vacuum-filtered via filter paper. The ethanol in the extract was evaporated in a rotatory evaporator (40 °C) [26]. Then, the resulting concentrate was lyophilized, resulting in a dried extract (Figure 1), whose weight was quantified to calculate the final yield considering the initial flour mass.

### 2.3. Extract Chemical Characterization

#### 2.3.1. Total Polyphenols and Antioxidant Capacity

The analysis of total polyphenols was determined in the dried extract by the Single-Ciocalteau assay [27]. The absorbance was measured (760 nm) and total polyphenols were expressed as grams of gallic acid equivalent (GAE) per 100 g of wet weight sample.

The antioxidant capacity was determined by the radical scavenging activity assay using DPPH (1,1-diphenyl-2-picrylhydrazyl) [28]. Briefly, lyophilized black corn extract (100 µL) was added to an ethanolic DPPH solution and stirred by vortex (3000 rpm/30 s). After incubation (30 min), the absorbance was measured (517 nm) and the DPPH radical scavenging activity was calculated as:

(1) $$\mathrm{Scavenging}\;\%=\frac{\mathrm{Ablank}-\mathrm{Asample}\;}{\mathrm{Ablank}}$$

#### 2.3.2. Anthocyanin Profile Analysis

The black corn anthocyanin-rich extract was analyzed by High Performance Liquid Chromatography (HPLC) Alliance Waters^®^ model 2690/5, with a Waters^®^ photodiode array detector model 2996 (scanning from 210 to 600 nm with quantification at 520 nm). The chromatographic separation was performed using a Thermo Hypersil BDS (Thermo Fisher Scientific, Waltham, MA, USA) C_18_ column (100 mm × 4.6 mm × 2.4 µm) at 40 °C, an injection volume of 20 µL, a total run time of 20 min, and a 1.0 mL min^−1^ flow rate. The mobile phase used was an aqueous solution of formic acid (Phase A) and acetonitrile (Phase B). The quantification was performed by using external standards. The gradient elution was 20% solvent B over 3 min, followed by a linear gradient up to 30% solvent B within 15 min and held there for 2 min, and then a linear gradient up to 60% solvent B in 13 min and held there for 2 min. Returning to initial conditions, 20% solvent B in 5 min and held there for 8 min for column rinse and re-equilibration. Mobile phase 2 consisted of an aqueous solution of formic acid as solvent A and acetonitrile as solvent B. The gradient was linear from 5 to 10.5% solvent B over 7 min 30 s and held there for 4 min 30 s, then a linear gradient up to 12% solvent B over 1 min, then another linear gradient up to 14% solvent B over 1 min, and then reduced back to 5% solvent B at 2 min 30 s and held there for 3 min 30 s for column rinse and re-equilibration [29]. Cyanidin-3-glucoside and pelaronidin-3-O-glucoside were used as standards.

### 2.4. Intra-Amniotic Experiment

Forty Cornish-cross fertile eggs from a commercial hatchery (Moyer’s chicks, Quakertown, PA, USA) were incubated under controlled temperature (37 ± 2 °C) and humidity (89 ± 2% humidity) in a poultry farm incubator at Cornell University Animal Science. All experimental procedures were carried out in accordance with the Cornell University International Animal Care and Use Committee (IACUC, protocol code: 2020-0077).

The black corn extract and the cyanidin-3-glucoside (C3G) were diluted in 18 MΩ H_2_O to verify the concentration to achieve an osmolarity value (Osm) of <320 Osm [18,22], in order to certify that the viable embryos would not be dehydrated upon the administration of the amniotic fluid. During the embryonic development (a total of 21 days), on day 17 of incubation, eggs with viable embryos (*n* = 36) were distributed by randomization into four groups with a similar weight frequency distribution. The groups were distributed as follows: No-injection (*n* = 7); H_2_O injection, 18 MΩ H_2_O (*n* = 8); BCE, 5% black corn extract in 18 MΩ H_2_O (*n* = 8); and C3G, 0.38% cyanidin-3-glucoside in 18 MΩ H_2_O (*n* = 8).

The intra-amniotic administration of black corn extract (1 mL/animal) was prepared at a concentration of 5% in accordance with our previous study [18]. The C3G was administered at a concentration of 0.38%, as this compound has yet to be tested intra-amniotically; hence, we chose to proceed with a lower dosage. A 1 mL solution was administered using a 21-gauge needle into amniotic fluid following candling [22,25]. Afterward, cellophane tape was used to seal the injection holes, and all the eggs were allocated to hatching baskets to minimize bias related to allocation. On day 21, after hatching, chickens were weighed and then euthanized by CO_2_ exposure, and the blood was collected by cardiac puncture. The duodenum and cecum were immediately collected, and part of the duodenum and cecum were immersed in liquid nitrogen and then kept at −80 °C until further analysis. Meanwhile, the other portion of the duodenum was fixed in a 10% (*v*/*v*) formalin solution for histological analysis.

### 2.5. Total RNA Extraction from Duodenum

Total RNA extraction from the proximal duodenum (*n* = 5 animals/group) was performed with a RNeasy Mini Kit, Qiagen Inc. (Cat # 74004, Valencia, CA, USA), as suggested by the manufacturer’s protocol. The procedures were performed under RNase-free conditions, and RNA was quantified by absorbance (260/280 nm). The integrity of the 18S ribosomal RNAs was carried out using agarose gel electrophoresis (1.5%) and staining with ethidium bromide. Extracted RNA samples were frozen at (−80 °C) until further analysis.

### 2.6. Gene Expression Analysis

The gene expression of the duodenum was determined by real-time polymerase chain reaction (RT-PCR) as described earlier [18,25]. Briefly, cDNA was created with a total of 20 μL of reverse transcriptase (RT) reaction completed in a BioRad C1000 touch thermocycler using the Improm-II Reverse Transcriptase Kit (Ca # A1250; Promega, Madison, WI, USA). The cDNA obtained was analyzed by Nanodrop (Thermo Fisher Scientific, Waltham, MA, USA). The concentration of cDNA was verified by measuring the absorbance (260/280 nm) with an extinction coefficient of 33 (for single-stranded DNA). The forward and reverse primers and the tested genes’ descriptions were designed based on the Genbank database, using Real-Time primer Design Tool software (IDT DNA, Coralvilla, IA, USA) (Table 1).

Real-time PCR amplifications were carried out under specific conditions: 95 °C (30 s followed by 40 cycles (95 °C, 15 s), annealing temperature for 30 s, and elongation at 60 °C for 30 s in the Bio-Rad CFX96 Touch (Hercules, CA, USA). The gene expression data was obtained as the lowest cyclic product (Cp) values based on the “second derivative maximum” as computed by Bio-Rad CFX Maestro 1.1 (Version 4.1.2433.1219, Hercules, CA, USA). The assays were quantified through a standard curve in the real-time qPCR analysis, and a 1:10 dilution prepared a standard curve with four points. The software procedure a Cp vs. log 10 concentration graph, and the efficiencies were calculated as 10 (1/slope). The specificity of the amplified real-time RT-PCR procedures was verified by melting curve analysis (60–95 °C) after 40 cycles, resulting in several different specific products with specific melting temperatures.

### 2.7. Intestinal Content and DNA Isolation

The cecum (*n* = 5 animals/group) from a separate chicken was aseptically removed and treated as shown elsewhere [18,30]. In short, the cecum content (200 mg) was placed into a plastic tube with phosphate-buffered saline (PBS) solution and homogenized through a vortex with glass beads (3 mm in diameter) for 3 min. To remove the debris, it was centrifuged, and the supernatant was collected. Before DNA extraction, the pellet was washed twice with PBS and stored at −20 °C. In order to perform the purification of DNA, the pellet was re-suspended in 50 mM ethylenediaminetetraacetic acid (EDTA) and treated with lysozyme (Sigma Aldrich Co., St. Louis, MO, USA). The bacterial genomic DNA was isolated using the Wizard Genomic DNA purification kit (Cat # A1120, Promega Corp., Madison, WI, USA).

### 2.8. Primers Design and PCR Amplification of Bacterial 16S rDNA

Primers for *Lactobacillus*, *Bifidobacterium*, *Clostridium*, *Escherichia coli*, and *L. planetarium* were used. The universal primers were designed based on prior research [25,30,31]. PCR products were separated by electrophoresis on a 2% agarose gel, stained with ethidium bromide, and quantified by Quantity One 1-D analysis software (Version 4.6.8, Bio-Rad, Hercules, CA, USA). All products were expressed relative to the content of the universal 16s rRNA primer product and the proportions of each examined bacterial group.

### 2.9. Histological Analysis

Duodenal morphology was performed as previously described [18,32]. Briefly, duodenum sections were fixed using buffered formaldehyde solution 4% (*v*/*v*), dehydrated, cleared, and embedded in paraffin. Sections (5 μm) were added to glass slices, deparaffinized in xylene, rehydrated in ethanol, and stained with Alcian blue/Periodic acid–Schiff. The morphometric measurements of villus height (μM), villus surface (μM), depth of crypts (μM), goblet cell number, and goblet cell diameter (μM) in the crypt and the villi, Paneth cell number, and Paneth cell diameter were assessed using a light microscope (CellSens Standard software, Olympus, Waltham, MA, USA). Five segments of each biological sample (*n* = 3/treatment group) were assessed, and ten randomly selected villi and crypts were analyzed per segment (50 replicates per biological sample). Villus surface area was obtained by the equation:

(2) $$\mathrm{Villus}\;\mathrm{surface}\;\mathrm{area}=2\;\frac{VW}{2}\;\times \;VL\;$$

where *VW* = villus width average of three measurements, and *VL* = villus length.

### 2.10. Statistical Analysis

Experimental groups were completely randomized. Statistically significant differences between experimental groups were conducted by a one-way Analysis of Variance (ANOVA) and a post-hoc Duncan test for those with a normal distribution. The mean for a normal distribution is tested using the Shapiro–Wilk normality test. The means without normal distribution were analyzed using Kruskal–Wallis and a post-hoc Dunn’s test. Data were expressed as mean ± standard error deviation (SED) and differences were considered significant when *p* < 0.05. The association and significance between intestinal biomarkers, bacterial population, and histological parameters were analyzed by Spearman’s rank correlation coefficient. GraphPad Prism^®^ version 8.0 software packages (GraphPad Software Inc., San Diego, CA, USA) were used for graphing and data analysis.

## 3. Results

### 3.1. Black Corn Extract Characterization

The cyanidin-3-glucoside (C3G) was identified as the principal anthocyanin constituent of black corn extract (BCE), followed by pelargonidin-3-O-glucoside. The BCE showed a high concentration of total phenolic compounds (555 mg GAE/100 g), and the antioxidant capacity was 70.79% (Table 2).

### 3.2. Effect of BCE on the Bacterial Population on Cecum Content

**Figure 2:**
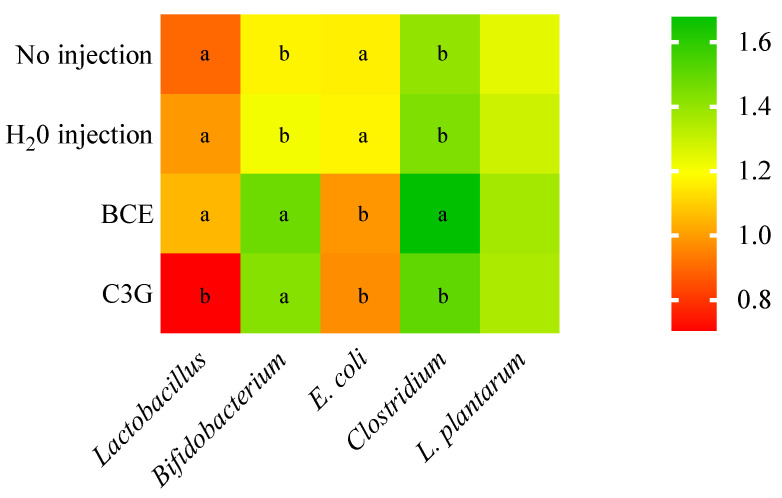
The effect of intra-amniotic black corn extract administration on the bacterial population from cecal content. The relative abundance is expressed in arbitrary units (AU). Values are means ± SED, *n* = 5 animals/group. BCE: black corn extract; C3G: cyanidin-3-glucoside. The treatment groups not indicated by the same letter are different (*p* < 0.05) by the post-hoc Duncan test. Squares without any letters: no difference among the treatments (*p* > 0.05).

The BCE promoted significant changes in the cecum bacterial populations. Specifically, the BCE and the G3G increased (*p* < 0.05) *Bifidobacterium* and decreased (*p* < 0.05) *E. coli* populations compared to No injection and H_2_O injection. The BCE group had the highest abundance of *Clostridium* compared to the other treatment groups. Further, the abundance of *Lactobacillus* significantly (*p* < 0.05) decreased after the C3G intra-amniotic administration compared to the control and BCE groups. The abundance of *L. plantarum* was similar (*p* > 0.05) among all experimental groups (Figure 2).

### 3.3. Effect of BCE on Duodenal Gene Expression

**Figure 3:**
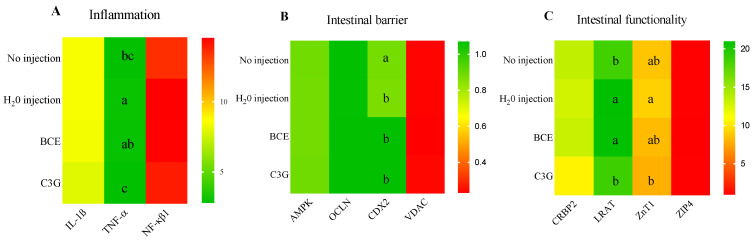
Effect of intra-amniotic administration of black corn extract on duodenal gene expression related to (**A**): intestinal inflammation biomarkers; (**B**): intestinal barrier biomarkers; (**C**): intestinal functionality biomarkers. Values are means (AU: arbitrary units) ± SED, *n* = 5 animals/group. BCE: black corn extract; C3G: cyanidin-3-glucoside. The treatment groups not indicated by the same letter are different (*p* < 0.05) by the post-hoc Duncan test. Squares without any letters: no difference among the treatments (*p* > 0.05). TNFα: tumor necrosis factor-alpha; NF-κβ1: nuclear factor kappa beta-1; IL-1β: interleukin 1 beta; AMPK: AMP-activated protein kinase; OCLN: occludin; CDX2: caudal-related homeobox transcriptional factor 2; VDAC: voltage-dependent anion channel; CRBP2: cellular retinol-binding protein-2; LRAT: lecithin retinol acyltransferase; ZnT1: Zinc transporter 1.

The gene expression of duodenal interleukin one beta (IL-1β) and nuclear factor kappa beta (NF-κβ) was similar (*p* > 0.05) among the experimental groups. The pro-inflammatory cytokine tumor necrosis factor-alpha (TNFα) was downregulated (*p* < 0.05) in the C3G group compared to BCE and the H_2_O injection (Figure 3A). Furthermore, to evaluate the intestinal physical barrier integrity, the mRNA expression of AMP-activated protein kinase (AMPK), occludin (OCLN), and voltage-dependent anion channel (VDAC) were determined, but no significant difference (*p* > 0.05) was observed among the groups for these variables. On the other hand, the caudal-related homeobox transcriptional factor 2 (CDX2) gene expression was downregulated (*p* < 0.05) after the intra-amniotic administration of H_2_O, BCE, and C3G compared with No injection (Figure 3B).

Intestinal functionality was assessed through intestinal transporters. The mRNA expression of cellular retinol-binding protein-2 (CRBP2) and ZIP 4 was similar among all experimental groups (*p* > 0.05). However, lecithin: retinol acyltransferase (LRAT) and zinc transporter 1 (ZnT1) downregulated (*p* < 0.05) in the C3G group compared to the H_2_O injection group, but there was no difference between the intra-amniotic administration of BCE and H_2_O for these markers (Figure 3C).

### 3.4. Effect of BCE on Duodenal Morphology

A morphological analysis of the duodenum was performed to observe the intra-amniotic effects of BCE in the duodenal mucosa. The animals that received the BCE had no changes in the villi height compared to the H_2_O injection (*p* > 0.05). The C3G group showed the highest villi height among all experimental groups (p < 0.05). Further, the duodenal depth crypt and the Paneth cell number were higher in the C3G compared to the BCE group (*p* < 0.05). The Paneth number was higher (*p* < 0.05) in the BCE when compared to the No injection (Table 3).

**Figure 4:**
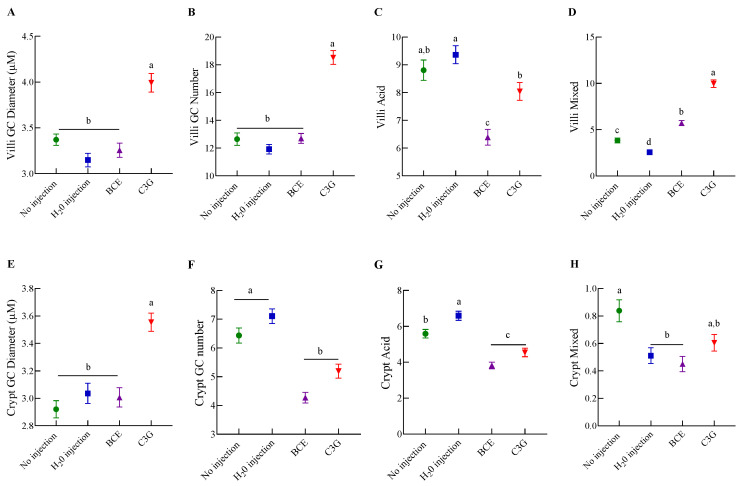
Effect of intra-amniotic administration of black corn extract on goblet cells: (**A**–**D**): villi goblet cell characteristics; (**E**–**H**) crypt goblet cell characteristics. Values are means ± SED, *n* = 3 animals/group. Treatment groups not indicated by the same letter are different (*p* < 0.05) by Kruskal–Wallis and a post-hoc of Dunn’s test. Mixed goblet cells are acidic and neutral. BCE: black corn extract; C3G: cyanidin-3-glucoside; GC: goblet cell.

Moreover, goblet cell (GC) morphological analysis was performed in the villi and the crypt. In the villi, the GC diameter (Figure 4A) and number (Figure 4B) were higher (*p* < 0.05) after the C3G administration intra-amniotically compared to the BCE group, which had similar values to the control groups. Furthermore, the BCE promoted a decrease (*p* < 0.05) of acid GC compared to the C3G, H_2_O injection, and No injection (Figure 4C). The villi mixed GC was higher in the BCE and C3G than in the H_2_O injection and No injection (Figure 4D). In the same way, in the crypt, the C3G increased (*p* < 0.05) the GC diameter compared to the other experimental groups, and BCE was similar to the control groups (Figure 4E). Further, the BCE and the C3G promoted a decrease in the GC number compared to the other groups (Figure 4F). After classifying the GC, we observed that the BCE and C3G have the lowest number of villi acid GC compared to the control groups (Figure 4G). There was no difference in the crypt mixed GC in the BCE group compared to the H_2_O injection and C3G (Figure 4H).

**Figure 5:**
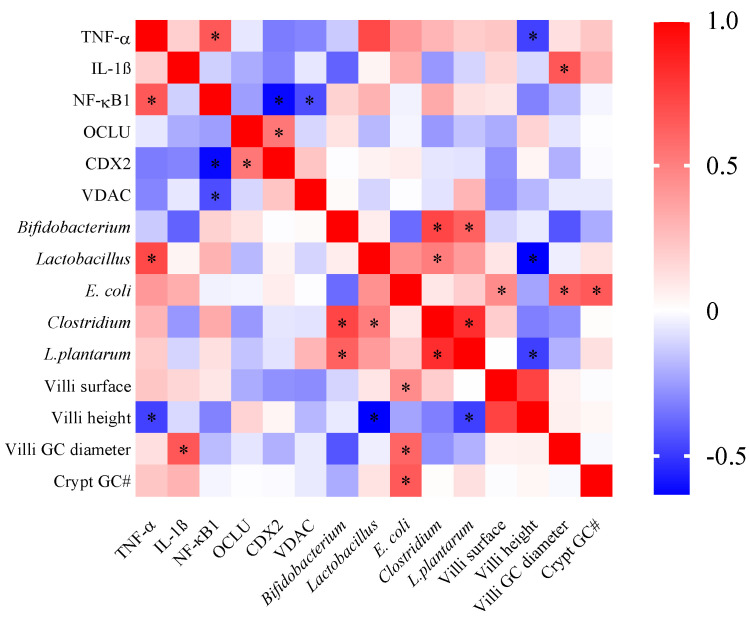
Correlation between intestinal biomarkers, bacterial population, and histological parameters. Colors from blue to red represented the *p*-value of the Spearman’s rank correlation coefficient. Blue: negative and red: positive correlation. * Significant correlation *p* < 0.05. TNF−α: tumor necrosis factor-alpha; NF−κB: nuclear factor kappa beta; IL−1β: interleukin 1 beta; MUC2: mucin 2; OCLN: occludin; CDX2: caudal-related homeobox transcriptional factor 2; VDAC: voltage-dependent anion channel. GC: goblet cells; #: number.

In our results, significant intestinal correlations were observed between the intestinal parameters investigated (Figure 5). Positive correlations were observed between *Bifidobacterium* and *Clostridium*, *E. coli* and villi GC diameter, and CDX2 and OCLU. Furthermore, villi height, TNFα, NF-κB1, and CDX2 showed a negative correlation.

## 4. Discussion

The current scientific literature suggests that the dietary intake of bioactive components offers significant health-promoting benefits [5,15]. Bioactive components include a range of phenolic components, in which each subgroup exerts different tissue and/or cellular effects and promotes beneficial responses in the organism [15,20]. Our study focused on the effects of black corn (*Zea mays*) anthocyanin-rich extract on intestinal functionality, morphology, and microbial populations in an intraamniotic approach. The intraamniotic administration of black corn extract (BCE) promoted a significant improvement in cecal *Bifidobacterium*, *Clostridium,* and reduced *E. coli*. populations. BCE did not change the duodenal brush border membrane morphology and functionality compared to the control groups.

The BCE composition showed significant levels of C3G and total phenolic compounds. Purple corn flour has shown an amount of anthocyanin (mg cyanidin-3-glucoside/100 g) varying from 220 [33] to 310.04 mg [24]. A wide variation has also been observed among different genotypes, from 12.8 to 93.5 mg C3G/g in 20 different genotypes [34]. In this context, considering our previous study with the same food source but as a flour (black corn flour) [13], the values of C3G in the extract (283.91 mg/100 g) were almost ten-fold higher relative to the flour (30.40 mg/100 g). Total phenolic compounds were similar in the extract (555 mg GAE/100 g sample) compared to the flour (614.30 mg GAE/100 g). Solid-liquid extraction with solvents is the simplest and most common method for extracting phenolic compounds, which is performed to achieve higher yields of the required compounds [35]. Polyphenolic compounds are secondary metabolites of plants that have an effect on plant adaptation to the environment [36], as well as potential bioactivities in animal organisms [15]. According to their chemical structure, phenolic compounds are classified into categories, in which the largest group is the flavonoids, with anthocyanin as a subgroup [37].

Furthermore, the 16s rDNA analysis investigated five bacterial populations and revealed that BCE and C3G increased *Bifidobacterium* and reduced *E. coli* populations in comparison to the other experimental groups (No injection and H_2_O injection). The C3G metabolism promotes the proliferation of the genus *Bifidobacterium* in the cecum [38]. Species of *Bifidobacterium* can produce a β-glucosidase enzyme, which supports the hydrolysis of C3G into aglycones and phenolic compounds, which in turn promotes the growth of these beneficial bacteria [17]. In a study with berries, the bifidogenic effect was attributed to the content of anthocyanin but also of polyphenols, as polyphenols contribute to creating a redox environment beneficial to the *Bifidobacteria* selection, which is favorable by a low oxidation-reduction potential [39,40]. Furthermore, the *E. coli* genus contains diverse pathogenic strains that may impair the epithelial barrier by disrupting tight junction proteins [41]. The protective effect of anthocyanin on pathogenic bacteria might be through its intestinal metabolite protocatechuic acid [12], which has been shown to inhibit the growth of *E. coli* [42], which agrees with the observed reduction of *E. coli* abundance in the current study. In agreement, the inhibition of *E. coli* might be associated with the villi goblet cell (GC) diameter and crypt GC number, as indicated by the positive correlation between these variables. GC produces the most important substance in the mucus layer: mucin, which forms a gel barrier against pathogenic bacteria [43]. Therefore, we speculate that a reduction in *E. coli* due to the BCE contributes to maintaining the GC number as the control.

Moreover, the BCE administration increased the *Clostridium* and *Lactobacillus* populations in cecal content compared to the C3G administration. In addition to C3G, other phenolic compounds are found in the black corn extract, which might explain these findings. Several *Lactobacillus* strains use phenolic compounds as a carbon source, thus maintaining their growth besides being involved in the hydrolysis of phenolic compounds due to fermentation [44]. Polyphenols are suggested to exert a prebiotic-like effect by increasing the *Lactobacillus* populations [15], and strains of *Lactobacillus* are considered probiotics due to their immunomodulatory and anti-inflammatory actions, inhibition of bacterial toxins, and competition with pathogens [45]. Therefore, for further investigation, and in addition to the anthocyanin profile, the focus should also be on phenolic characterization.

In the present study, the BCE did not affect the intestinal brush border membrane (BBM) biomarkers: interleukin one beta (IL-1 β), tumor necrosis factor-alpha (TNFα), and nuclear factor kappa beta (NF-κβ). However, the isolated C3G administration downregulated TNFα expression compared to H_2_O injection and BCE. Considering the chemical composition of the BCE, it provided a higher administration of cyanidin-3-glucoside (0.014 mg/mL) compared to the isolated C3G, which provided an administration of 0.003 mg/mL. It was previously demonstrated that the effect of polyphenols to downregulate TNFα gene expression was concentration dependent, as 2% saffron extract downregulated TNFα expression, but 5 and 10% did not have this effect, as tested in vivo via intra-amniotic administration [21]. Additionally, we highlight that even with a high dosage of cyanidin-3-glucoside, the black corn extract did not exert any detrimental effect on the investigated inflammatory pathway, as there was no difference in the biomarkers in the BCE group versus the controls. Further investigation and other biomarkers are required to address phenolic compounds’ dosage and profile to exert an anti-inflammatory effect.

The BCE administration did not alter villi height and GC diameter (crypt and villi), relative to the H_2_O injection. However, these variables were lower in the BCE compared to the C3G. Therefore, we hypothesize that this result is not attributed to the anthocyanin level but probably to other phenolic components that might be present in the extract, such as protocatechuic, vanillic, p-hydroxycinnamic, and ferulic acid [46]. In agreement, the administration of saffron flower extract, a source of phenolic components, showed a dose-dependent effect on decreasing villus surface area, goblet cell number, and diameter [21]. The duodenal morphometric observations in the current study may indicate that depending on the polyphenolic components can exert distinct effects on the brush border development and absorptive capacity [21,23,34]. The variation of polyphenol composition in four distinct types of beans contributed to different results in intestinal morphology and functionality [23]. Interestingly, regarding the type of goblet cells, the BCE group showed the lowest number of acidic GC (in the villi and crypt). A luminal acidic pH facilitates the growth of beneficial bacteria over detrimental bacteria [47]. Therefore, the decrease of acidic GC might be associated with the increase in the *Clostridium* population verified in the BCE group [48]. However, even with the growth of *Clostridium* bacteria, the BCE did not affect Paneth cell number relative to the H_2_O injection groups. These cells indicate an early state of inflammation, infection, and toxicity due to the secretion of antimicrobial peptides [49].

Finally, in a prior experiment, we showed the beneficial effects of black corn soluble extract (composed of 6.33 g of total dietary fiber/100 g) on intestinal inflammation parameters, morphology, and BBM barrier function [18]. On the other hand, in the present study, the black corn extract (5%) is composed mainly of phenolic components without any dietary fiber. It modulates the cecal microbiome by changing specific bacterial populations and maintaining intestinal morphology and functionality without detrimental effects. Thus, we highlight the positive effects of black corn anthocyanin-rich extract without any soluble dietary fiber, which was able to improve the cecal microbial populations and maintain intestinal morphology and functionality without any detrimental effects in vivo.

## 5. Conclusions

The black corn anthocyanin-rich extract improved the cecal microbiome by increasing *Bifidobacterium* and *Clostridium*, reducing the *E. coli* population while maintaining intestinal morphology and functionality. Further, the C3G group showed additional effects on improving intestinal morphology versus the BCE, suggesting that the combination and dosage of phenolic compounds might interfere with intestinal morphology development. Therefore, our results suggest that black corn anthocyanin-rich extract is a promising target matrix to be used as a functional extract to improve intestinal microbial populations, and further studies in terms of dosage and profile of phenolic compounds in this food matrix are now warranted.

## Figures and Tables

**Table 1:** Sequence and description of experimental primers.

Analyte	Forward P. (5′-3′)	Reverse P. (5′-3′)	Base Pairs Length	GI Identifier
Inflammatory Response				
TNFα	GACAGCCTATGCCAACAAGTA	TTACAGGAAGGGCAACTCATC	109	53,854,909
NF-κB1	CACAGCTGGAGGGAAGTAAAT	TTGAGTAAGGAAGTGAGGTTGAG	100	2,130,627
IL-1β	CTCACAGTCCTTCGACATCTTC	TGTTGAGCCTCACTTTCTGG	119	88,702,685
Intestinal Functionality				
MUC2	CCTGCTGCAAGGAAGTAGAA	GGAAGATCAGAGTGGTGCATAG	272	423,101
OCLN	GTCTGTGGGTTCCTCATCGT	GTTCTTCACCCACTCCTCCA	124	396,026
AMPK	CTCCACTTCCAGAAGGTTACTT	GCAGTAGCTATCGTTCATCCTATC	140	427,185
CDX2	ACCAGGACGAAGGACAAATAC	CTTTCCTCCGGATGGTGATATAG	103	374,205
VDAC2	CAGCACTCGCTTTGGAATTG	GTGTAACCCACTCCAACTAGAC	99	395,498
18S rRNA	GCAAGACGAACTAAAGCGAAAG	TCGGAACTACGACGGTATCT	100	7,262,899

P: primers; TNFα: tumor necrosis factor-alpha; NF-κB1: nuclear factor kappa beta 1; IL-1β: interleukin 1 beta; MUC2: mucin 2; OCLN: occludin; AMPK: AMP-activated protein kinase; CDX2: caudal-related homeobox transcriptional factor 2; VDAC: voltage-dependent anion channel.

**Table 2:** Characterization of black corn anthocyanin-rich extract (BCE).

Components	Amount	Retention Time (min)
Cyanidin-3-glucoside (mg/100 g)	283.91	6.5
Pelargonidin-3-O-glucoside (mg/100 g)	39.57	8.7
Total phenolic compounds (mg GAE/100 g sample)	555.00	-
DPPH (%)	70.79	-

GAE: gallic acid equivalent. Cyanidin-3-glucoside and pelargonidin-3-O-glucoside were quantified by High Performance Liquid Chromatography; total phenolic compounds and DPPH were analyzed by spectrophotometry.

**Table 3:** Effect of intra-amniotic administration of black corn extract on villi height, surface, and depth crypt.

	No Injection	H_2_O Injection	BCE	C3G
Villi height (μM)	193.12 ± 3.75 ^b^	171.50 ± 4.01 ^c^	169.69 ± 2.10 ^c^	202.43 ± 2.81 ^a^
Villi surface (μM^2^)	12,324.31 ± 344.23 ^b^	11,740.52 ± 336.59 ^b^	11,181.40 ± 224.95 ^b^	15,250.89 ± 390.56 ^a^
Depth crypt (μM)	24.98 ± 1.01 ^b^	37.30 ± 1.08 ^a^	24.78 ± 0.62 ^b^	35.35 ± 0.99 ^a^
Paneth cell number	0.95 ± 0.07 ^c^	1.30 ± 0.04 ^b^	1.27 ± 0.04 ^b^	2.49 ± 0.14 ^a^
Paneth cell diameter	1.69 ± 0.03 ^a^	1.55 ± 0.02 ^b^	1.50 ± 0.02 ^b^	1.58 ± 0.02 ^a,b^

Values are means ± SED, *n* = 3 animals/group. BCE: black corn extract; C3G: cyanidin-3-glucoside. Treatment group means for specific variables followed by the same letter are not significantly different (*p* > 0.05) by Kruskal–Wallis and a post-hoc Dunn’s test.

## Data Availability

Data available upon reasonable request.
